# Comparison of dose calculation approaches and clinical dose–response in image-guided head&neck brachytherapy of the oral cavity

**DOI:** 10.1016/j.ctro.2025.100968

**Published:** 2025-05-08

**Authors:** Sabrina Schaller, Vratislav Strnad, Claudia Schweizer, Dorota Lubgan, Ricarda Merten, Rainer Fietkau, Christoph Bert, Andre Karius

**Affiliations:** a Department of Radiation Oncology, Universitätsklinikum Erlangen, Friedrich-Alexander-Universität Erlangen-Nürnberg (FAU), Erlangen, Germany; b Comprehensive Cancer Center Erlangen EMN (CCC Erlangen-EMN), Erlangen, Germany; c Translational Radiobiology, Department of Radiation Oncology, Universitätsklinikum Erlangen, Friedrich-Alexander-Universität Erlangen-Nürnberg (FAU), Erlangen, Germany

## Abstract

**Purpose:**

Model-based dose calculation algorithms (MBDCAs) are increasingly applied in brachytherapy, but their considerations in dose–response analyses is still lacking. This study aimed to assess correlations between both TG-43 and MBDCA dosimetry and reports on clinical outcomes for oral cavity brachytherapy.

**Methods:**

We considered 158 patients with oral cavity cancer treated in our institution between 2012 and 2021. Survival outcomes and toxicity (soft tissue necrosis, osteoradionecrosis, mucositis, xerostomia) were reported for a median follow-up of 80 months (2–152 months). All clinical, TG-43 based treatment plans were re-calculated using a MBDCA integrated into our planning system. Differences considering several target volume, tissue, and bone dose parameters were evaluated. Parameter correlations with clinical outcomes and thresholds associated with increased toxicity were investigated.

**Results:**

Cumulative local recurrence, soft-tissue necrosis, osteoradionecrosis, mucositis, and xerostomia rate were 21 %, 22 %, 28 % and 79 % considering all patients. Substantial differences between MBDCA and TG-43 were observed, especially regarding high-dose areas with changes up to 19 %. A number of dose-toxicity correlations were observed, as for osteoradionecrosis (1.6 % vs. 10.3 % for bone D_2ccm_ ≥ 59.3 Gy), soft tissue necrosis (16 % vs. 32 % for tissue D_5ccm_ ≥ 87.7 Gy), and local recurrence (13 % vs. 25 % for dose non-uniformity ratio < 0.29) (using the MBDCA). Target volumes ≥ 10.2–11.8ccm were associated with increased rates of soft tissue necrosis and mucositis. Dosimetric thresholds and correlations differed between TG-43 and MBDCA.

**Conclusion:**

For oral cavity brachytherapy, several important dosimetric thresholds associated with increased toxicity were determined. MBDCAs for corresponding dose calculations should be advanced and used in clinical practice.

## Highlights

• Osteoradionecrosis is correlated to bone D_2ccm_ ≥ 59.3 Gy.
• Soft tissue necrosis is correlated to tissue D_5ccm_ ≥ 87.7 Gy.
• Target volumes ≥ 10.2–11.8ccm are associated with increased rates of soft tissue necrosis and mucosits.
• Dose thresholds for toxicity differed between TG-43 and model-based dose calculation.
• Model-based dose calculation differed partly substantially from TG-43 dose calculations.

## Introduction

Interstitial brachytherapy is an established method for the treatment of tumors of the oral cavity, including mobile tongue and floor of mouth[1,2,3]. Depending on disease extent and stage, brachytherapy can be applied as sole modality as well as in combination with external beam radiotherapy (EBRT) or surgery, and several studies already reported excellent outcomes regarding local control and toxicity[4,5,6,7].

In this respect, many clinical studies paving the way for the establishment of modern head and neck brachytherapy were performed in the 1990s and early 2000s[8,9,10]. However, due to limited availability at that time, image-guided treatments based on cross-sectional imaging and three-dimensional (3D) treatment planning considering dose-volume histogram (DVH) parameters as conducted nowadays[1] were usually not addressed in these investigations. In addition, the previously frequently used low-dose-rate (LDR) regime became more and more obsolete[1], and model-based dose calculation algorithms (MBDCAs) as described in the AAPM TG-186 report[11] enabled dose calculations beyond the purely water-based TG-43 formalism[12]. Some clinical reports already suggested a reduced incidence of side effects as, e.g., soft tissue necrosis for image-guided pulsed- or high-dose-rate (PDR, HDR) oral cavity brachytherapy compared to the pre-3D era[8,13,14]. However, there is still a lack of corresponding dose–response analyses assessing the dependence of clinical outcomes on dose-volume parameters[15], especially considering large patient cohorts and MBDCAs.

The aim of this work was first to report clinical outcomes of 158 patients receiving image-guided brachytherapy of the oral cavity. Second, we assessed dosimetric differences between TG-43 and a MBDCA (considering both target volume and surrounding tissues) occurring for this anatomical region, and evaluated correlations between clinical outcomes and dose-volume parameters for both dose calculation approaches. This aimed to identify implant requirements associated with reduced toxicity and high treatment quality.

## Materials and Methods

### Clinical Workflow and patients

We retrospectively analyzed clinical and dosimetric data of altogether 158 patients (111 male, 47 female; median age: 63 years, range: 26–87 years) with at least one documented follow-up who received interstitial brachytherapy of the oral cavity at our institution from 2012 to 2021. Brachytherapy was administered either as sole treatment without tumor resection or any kind of EBRT (38/158 patients, 24 %), as sole brachytherapy after tumor resection (82 cases, 52 %), or as boost following definitive EBRT without prior tumor resection (38 cases, 24 %). The most important patient characteristics are summarized in Table 1.

**Table 1:** Listed are the patient characteristics, tumor histology and localization for all examined patients. The percentage numbers were calculated with respect to the total cohort size of 158 patients considered. Tumor classification is provided according to the Union for International Cancer Control (UICC) for each patient. T1-4 report the tumor extension, N the lymph node involvement (N0: no involvement; N+: any involvement), G the grading, L the lymphatic vessel invasion (L0: no invasion; L1: invasion), and R the completeness of operation (R0: complete resection; R+: any incomplete resection).

*Parameter*	*Number of patients*	*Patient fraction [%]*
*Age (Median: 63 years, range: 26*–*87 years)*		
*< 60*	*61*	*38.6*
*≥ 60*	*97*	*61.4*
*Gender*		
*Male*	*111*	*70.3*
*Female*	*47*	*29.7*
*Localization*		
*Floor of Mouth*	*41*	*25.9*
*Mobile Tongue*	*117*	*74.1*
*Histology*		
*Squamous cell carcinoma*	*153*	*96.8*
*Other*	*5*	*3.2*
*Tumor classification*		
*T1*	*54*	*34.2*
*T2*	*75*	*47.5*
*T3*	*21*	*13.3*
*T4*	*8*	*5.1*
*Lymph node involvement*		
*N0*	*127*	*80.4*
*N+*	*31*	*19.6*
*Grading*		
*G1-2*	*90*	*57.0*
*G3-4*	*68*	*43.0*
*Lymphatic vessel invasion*		
*L0*	*147*	*93.0*
*L1*	*11*	*7.0*
*Resection state*		
*No tumor resection*	*76*	*48.1*
*R0*	*71*	*44.9*
*R+*	*11*	*7.0*
*Treatment modality*		
*Sole brachytherapy*	*38*	*24.1*
*Brachytherapy + External beam radiotherapy*	*38*	*24.1*
*Adjuvant brachytherapy after surgery*	*82*	*51.9*

**Fig. 1:**
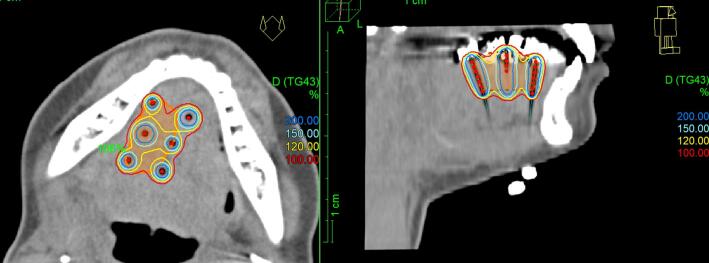
Show is a graphical illustration of the implant geometry considered in the present work. The orange area refers to the target volume, and the isodose lines are indicated as shown in the graphic in percentage of the prescribed dose. Graphic created with Oncentra Brachy.

The insertion of plane-parallel running plastic catheters for brachytherapy (Fig. 1) was conducted under nasotracheal general anesthesia for all patients in cooperation with our ENT surgery department. The implantation was guided by palpation and preoperative imaging (computed tomography (CT) or magnetic resonance imaging (MRI)), as described in detail by Strnad et al.[8]. The clinical target volume (CTV) comprised, depending on the case, the macroscopic tumor or tumor bed with a safety margin of 10 mm in all directions, excluding mandible and skin. Afterwards, a CT scan with 2 mm slice thickness was acquired for treatment planning, on which catheters were reconstructed and mandible as organ at risk (OAR) as well as CTV were contoured within the Oncentra Brachy planning system (Nucletron, Netherlands). Dwell positions and times of a 192-Ir afterloader source were manually defined and optimized to ensure an optimal CTV coverage V_100,CTV_ while simultaneously minimizing excessive dose hotspots (i.e., the 150 % isodose volumes ϑ_150_) and keeping the dose non-uniformity ratio[16] DNR= ϑ_150_/ ϑ_100_ < 35 %. Note that no bite block or spacer for distancing the hard palate from the oral tongue or floor of mouth was applied during the entire treatment course.

The treatment was delivered either in the PDR (128 patients) or HDR (30 patients) regime, depending on clinical availability. In case of PDR, hourly irradiations for 24 h per day with median physical pulse-doses of 0.5 Gy (range: 0.3–0.63 Gy) up to a total dose of 12 Gy (8–26 Gy; brachytherapy after EBRT) or 50 Gy (30–64 Gy; brachytherapy without EBRT) were performed. HDR treatments were conducted twice daily with median fraction doses of 3.4 Gy (2.6–4.5 Gy) up to 16 Gy (10–27 Gy; after EBRT) or 40.8 Gy (29–54 Gy; without EBRT). A median EBRT prescription dose of 56 Gy (30–68 Gy) was applied.

### Dose calculation approaches

Brachytherapy dose calculations in clinical routine were performed using the TG-43 formalism for all patients, which represents the current standard in this respect and assumes a pure water environment around the source[12].

To explicitly consider also air, bones, and dental implants during dose calculation in the present study, all clinical treatment plans were re-calculated with the Advanced Collapsed Cone Engine (ACE) representing the MBDCA integrated into Oncentra Brachy (version 4.6.2.). The underlying ACE algorithm was described in detail previously[17,18,19,20] and required knowledge about the material/tissue compositions present within the treatment region. For this reason, the CTV-surrounding normal tissue, air, and all bones were additionally contoured on the planning-CT. A dentist identified and delineated any teeth, bone/teeth implants, dentures, and metal present. All structures were then assigned to the respective tissue and material types listed in the TG-186 recommendation[11] or ICRU 46 report[21], referring to the materials also selectable in Oncentra Brachy. ACE calculations were then performed assuming uniform materials (hereafter named ACE_uniform_), i.e. without taking exact Hounsfield-units of each voxel into account to reduce the impact of image artifacts on dose calculations.

The physical dose distributions obtained by ACE_uniform_ and TG-43 were converted into doses biologically isoeffective to fractionated irradiations with 2 Gy single doses (EQD2) following the linear-quadratic model[22,23]. In this respect, an alpha/beta ratio and repair half-life of 3 Gy and 3.2 h for both CTV and normal tissue[24,25,26] as well as 0.85 Gy and 1.5 h for bones[24,27,28] was assumed. This conversion aimed at a comparability of different doses and treatment regimens regarding the clinical outcome assessment.

### Clinical outcomes and dosimetric impact

At each follow-up, treatment-related toxicity was documented considering the Radiation Therapy Oncology Group (RTOG) and European Organization for Research and Treatment of Cancer (EORTC) criteria[29]. Based on this, the cumulative incidence of soft tissue necrosis, osteoradionecrosis, mucositis, and xerostomia was calculated. To adequately capture late toxicity events, only patients with a follow-up ≥ 3 months were considered for this purpose. The impact of treatment concept and dose rate regime was investigated using Kaplan-Meier estimators and logrank tests[30]. Similarly, overall survival (OS), cumulative local recurrence rate (CLRR), and disease-free survival (DFS) were evaluated for all 158 treated patients.

For the dosimetric assessment, we considered the isodose volumes ϑ_200_, ϑ_150_, ϑ_100_, and ϑ_85_, the DNR, as well as the doses delivered to normal tissue (D_2ccm_, D_5ccm_, D_7.5ccm_), bones (D_0.1ccm_, D_2ccm_, D_5ccm_), and CTV (D_75_, D_90_, D_98_, V_100,CTV_). D_x_ and D_xccm_ refer to the doses the most exposed x% and x ccm of a structure received, respectively. Absolute differences in these EQD2 doses between ACE_uniform_ and TG-43 were determined (we calculated ACE_unifom_ minus TG-43) next to relative deviations ensuring comparability of results independent of differences in target volumes or treatment doses.

To assess the impact of dosimetry on clinical outcomes, the examined patients were separated into cohorts by determining for each evaluated dose-volume parameter and the CTV volume a specific threshold associated with the largest statistical power. This was done based on Barnard’s test[31] used to evaluate the significance of the cohort separations. Note that for patients receiving EBRT, all aforementioned evaluated dose parameters were determined from the brachytherapy treatment plan and added to the corresponding doses prescribed in the EBRT treatment plan (both converted into EQD2 as mentioned above). Exceptions were the isodose volumes and the DNR, which were analyzed for brachytherapy exclusively. Decreasing/increasing monotonic dependencies of outcomes on dosimetric results (independent of separating cohorts) were investigated using binary logistic regression and Wald-tests[32]. The significance level of all statistical tests was 5 %.

## Results

### Dosimetry

**Fig. 2:**
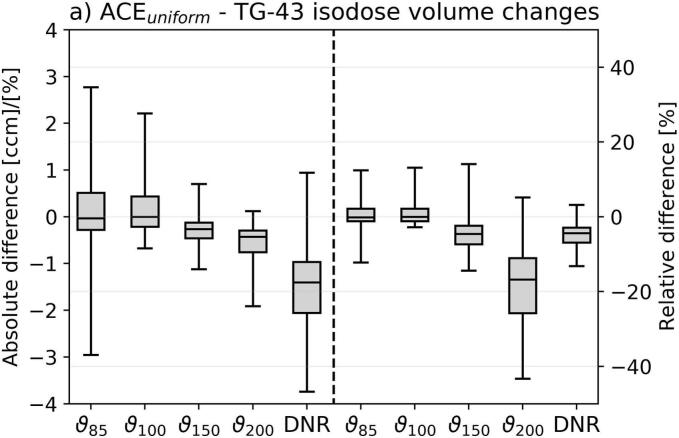

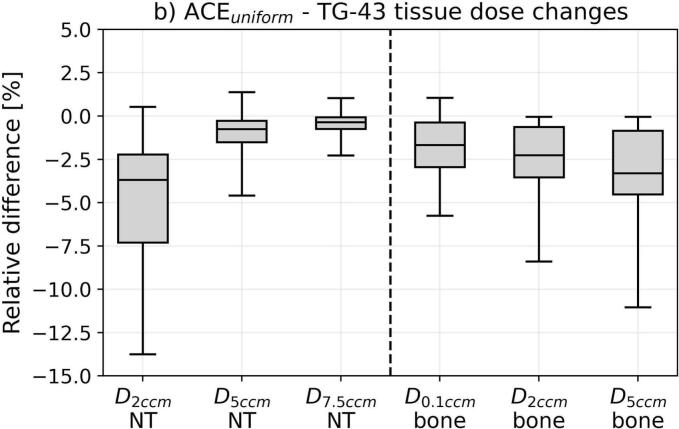

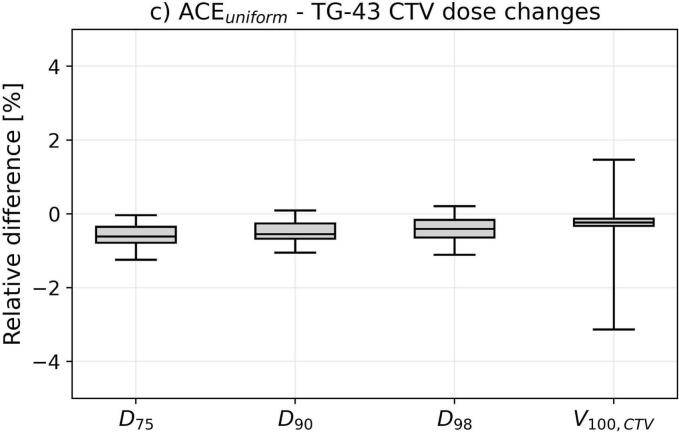
Shown are the dosimetric deviations between ACE_uniform_ and TG-43 dose calculations. For the isodose volume changes (a), both absolute differences (in ccm for the ϑ_200_, ϑ_150_, ϑ_100_, and ϑ_85_ and percentage points for the dose non-uniformity ratio DNR) as well as relative differences were provided. For tissue (b) and CTV (c) dose changes, the relative deviations were calculated to ensure comparability independent of differences in target volumes or treatment doses. In the boxplots, the horizontal lines indicate the median, the boxes the interquartile ranges, and the whiskers the 95th percentile of the results. Outliers were not shown for clarity of illustration. Abbreviation: NT normal tissue.

Comparing ACE_unifom_ to TG-43 revealed partly strong differences in the investigated dosimetric parameters (Fig.2). Considering the isodose volumes, ϑ_200_ deviated by −0.6 ± 0.5 ccm (mean ± standard deviation; range: −3.1–0.4 ccm) between ACE_unifom_ and TG-43, corresponding to relative deviations of –19 ± 12 % (−58–19 %). Model-based dose calculation thus led to reductions of high-dose areas. For ϑ_150_, ϑ_100_, and ϑ_85_, absolute deviations of −0.3 ± 0.4 ccm, 0.2 ± 0.8 ccm, and 0.1 ± 1.2 ccm, referring to relative differences of −4 ± 6 %, 1 ± 4 %, and 1 ± 5 % with ranges of −18–20 % (ϑ_150_), −16–15 % (ϑ_100_), and –19–15 % (ϑ_85_), were found, respectively. These observations were concomitant with relative DNR alterations of −5 ± 4 % (−16–7 %). Thus, depending on the specific patient case, varying the dose calculation approach was associated with a partly substantial relative change of the isodose volumes.

Similar observations were made for normal tissue, which revealed absolute D_2ccm_, D_5ccm_, and D_7.5ccm_ changes of −8 ± 10 Gy (−64–15 Gy), −1 ± 2 Gy (−8–17 Gy), and −0.2 ± 0.4 Gy (−2.2–1.1 Gy) for the entire brachytherapy course, respectively. This referred to relative differences of −5 ± 4 % (−25–6.3 %), −1 ± 2 % (−6–3 %), and −0.4 ± 0.8 % (−4–2 %). D_2ccm_ was largely affected by high-dose areas associated with catheters located outside the CTV and thus decreased using ACE_uniform_ similar to the abovementioned high-dose volumes. In contrast, D_5ccm_ and D_7.5ccm_ comprising more extended regions with less high-dose impact yielded smaller alterations between ACE_uniform_ and TG-43. All evaluated bone parameters showed negative mean changes of −1 ± 2 % (D_0.1ccm_) to −3 ± 3 % (D_5ccm_), indicating that TG-43 typically overestimated the bone doses. It has to be mentioned that dosimetric variations were particularly more pronounced for regions close to bones and air-transitions, due to the corresponding lack of backscatter compared to the TG-43 assumption of a pure water environment.

For the CTV D_75_, D_90_, D_98_, and V_100,CTV_, we found only small relative variations of −0.6 ± 0.4 % (−1.7–1.3 %), −0.5 ± 0.4 % (−1.4–1.4 %), −0.4 ± 0.5 % (−3–2 %), and −0.3 ± 1.0 % (−6–4 %). Hence, the evaluated target volume parameters were limited affected by choosing the dose calculation approach.

### Efficacy

**Fig. 3:**
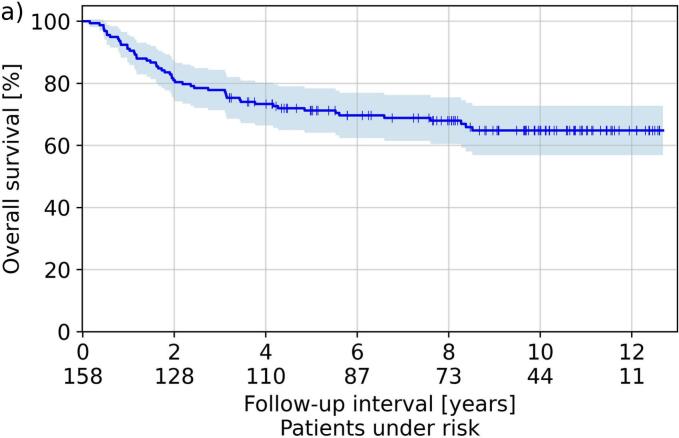

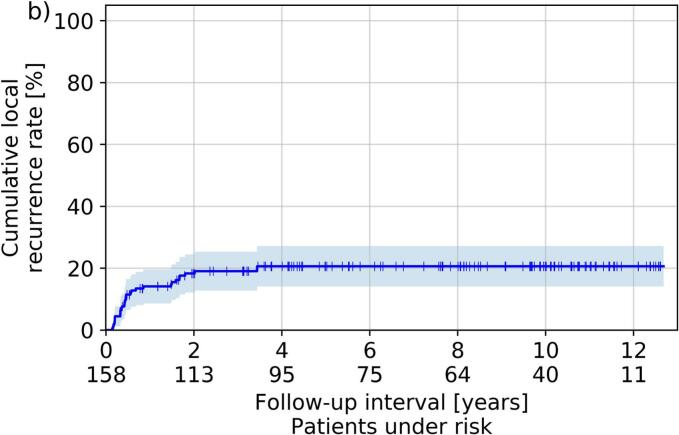

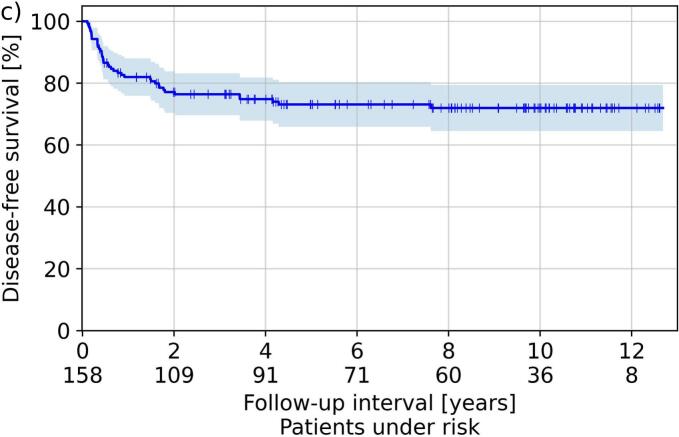
Shown in Kaplan-Meier style are the respective estimators obtained for overall survival (a), cumulative local recurrence rate (b), and disease-free survival (c). Shadowed areas refer to the 95th percentage confidence intervals.

The median follow-up was 80 months (2–152 months) and resulted in the Kaplan-Meier curves for OS, CLRR, and DFS illustrated in Fig.3. The Kaplan-Meier estimator for CLRR, DFS and OS, resulted to 14 %, 82 % and 91 % after one year, 18 %, 77 % and 81 % after two years, and 21 %, 73 %, and 71 % after five years, respectively. Considering all treated cases, a recurrence was clinically diagnosed in 31 (20 %) patients. Neither separation by treatment concept (brachytherapy after EBRT, sole brachytherapy, brachytherapy after tumor resection; five-year CLRR 20 %, 21 %, 20 %, respectively) nor by dose rate regime (PDR vs. HDR; five-year CLRR 20 % vs. 24 %) resulted in significant differences in the associated outcomes (p ≥ 0.2 and p > 0.06, respectively).

Considering dosimetric parameters as possible prognostic factor, the statistical analysis using Barnard’s test showed a significant decrease of cumulative local recurrence rates for patients with DNR values < 0.31 for TG-43 (p < 0.01; 11 % vs. 25 %) and < 0.29 for ACE_uniform_ (p = 0.03; 13 % vs. 25 %), indicating respective thresholds associated with increased recurrence rates for both dosimetry approaches. However, using logistic regression, a significantly increasing monotonic dependence of recurrences on the DNR was only found for TG-43 calculations (p = 0.04), but not for ACE_uniform_. The dose–response assessment was thus affected by the choice of the dose calculation procedure.

### Side effects

One of the 158 treated patients died two months after brachytherapy and was therefore not included in the toxicity analysis requiring at least one follow-up at ≥ 3 months (section "Clinical outcomes and dosimetric impact"). This analysis therefore included 157 patients with a median follow-up of 80 months (range: 4–152 months). Soft tissue necrosis, osteoradionecrosis, mucositis, and xerostomia of any grade were recorded for 34 (22 %), five (3 %), 44 (28 %), and 125 (80 %) of all treated patients, respectively. However, the cumulative incidence for grade 3 mucositis was only 8 %, whereas it was 4 % for grade 3 xerostomia. No grade 4 mucositis and xerostomia were observed. None of the patients diagnosed with osteoradionecrosis had previously undergone surgery on the mandible known to be an associated risk factor[33].

**Fig. 4:**
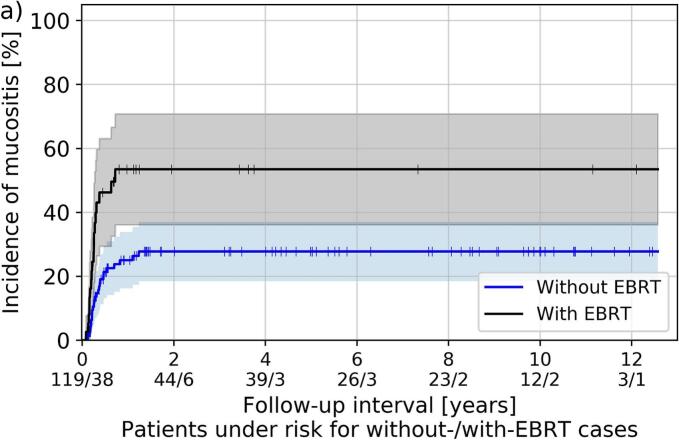

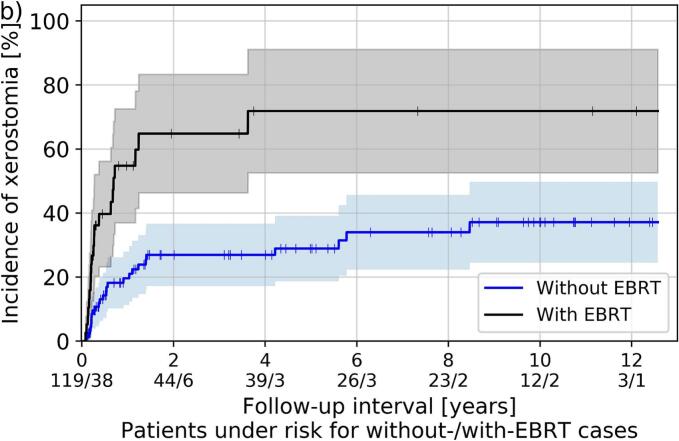
Kaplan-Meier estimator of mucositis (a) and xerostomia grade ≥ 2 (b) for patients included in the toxicity analysis and receiving brachytherapy as boost following EBRT (labeled “with EBRT”) and either definitive brachytherapy or sole brachytherapy after tumor resection (labeled “without EBRT”). Shaded areas refer to the 95th percentage confidence interval.

Separated by dose rate regime, no significant differences regarding the occurrence of examined side effects between HDR and PDR was found. Furthermore, no differences in soft tissue necrosis and osteoradionecrosis were found depending on the treatment concept. However, xerostomia and mucositis were increased in patients receiving EBRT compared to treatments without EBRT (p ≤ 0.03), as shown in Fig.4. Importantly, moderate to severe (i.e., grade ≥ 2) xerostomia was clinically diagnosed in 55 % (with EBRT) and 22 % (without EBRT) of the patients, whereas corresponding mucositis was observed in 50 % versus 21 % of all cases, respectively.

The significance of the evaluated dosimetric parameters considering TG-43 in relation to the incidence of side effects is summarized in Table 2. Explicit sizes of all examined isodose volumes as well as CTV volumes larger than 10.2–11.8 ccm were associated with increased rates of soft tissue necrosis (p < 0.01, 14 % vs. 32 % for CTV volumes below/above the determined threshold) and mucositis (p < 0.01, 16 % vs. 38 %). The values of D_2ccm_ ≥ 173.8 Gy, D_5ccm_ ≥ 85.4 Gy, and D_7.5ccm_ ≥ 58.2 Gy (TG-43) for the normal tissue of the oral cavity represented indicators for enhanced risk of soft tissue necrosis (up to 18 % vs. 34 %), mucositis (up to 21 % vs. 41 %), and xerostomia (up to 73 % vs 95 %) as well. The probability of osteoradionecrosis was significantly (p = 0.02, up to 1.6 % vs. 10.7 %) increased with D_2ccm_, D_1ccm_, and D_0.1ccm_ of the mandible exceeding 58.6–66.1 Gy (TG-43), whereby the small number of only five patients recorded with this toxicity has to be considered. However, an increasing monotonic relationship was only observed between xerostomia and normal tissue D_7.5ccm_ (p = 0.02) using logistic regression.

**Table 2:** Results of the dose–response analysis considering side effects. For each investigated parameter, the specific threshold grouping the patients with the largest statistical power (p-values p_B_) are shown in the “threshold” columns (first and second row represent results for TG-43 and ACE_uniform_, respectively). The “cohort” columns of form a/b – c/d indicated the patient numbers featuring dose values smaller (b) than and larger than or equal to (d) the threshold, and the number of patients (a and c) in which the corresponding side effects occurred. Significant results (p < 0.05) were marked bold. Abbreviations: NT normal tissue; B bone; vol. volume; DNR dose-nonuniformity ratio.

	Soft tissue necrosis			Osteoradionecrosis			Mucositis			Xerostomia		
Param.	Thresh.	Cohorts	P_B_	Thresh.	Cohorts	P_B_	Thresh.	Cohorts	P_B_	Thresh.	Cohorts	P_B_
ϑ_85_	24.5 ccm 27.4 ccm	10/81–24/76 13/94–21/63	**<0.01** **<0.01**	− −	− −	− −	23.3 ccm 27.4 ccm	14/75–30/82 19/94–25/63	**<0.01** **<0.01**	22.4 ccm 24.0 ccm	54/72–71/85 59/77–66/80	0.10 0.16
ϑ_100_	21.0 ccm 20.8 ccm	13/96 – 21/61 12/92 – 22/65	**<0.01** **<0.01**	− −	− −	− −	18.6 ccm 20.8 ccm	14/76 – 30/81 18/92 – 26/65	**<0.01** **<0.01**	17.5 ccm 18.7 ccm	55/73 – 70/74 58/76 – 67/81	0.10 0.13
ϑ_150_	6.3 ccm 5.7 ccm	12/91 – 22/66 10/80 – 24/77	**<0.01** **<0.01**	− −	− −	− −	6.3 ccm 5.7 ccm	19/92 – 25/65 15/80 – 29/77	**<0.01** **<0.01**	5.6 ccm 5.6 ccm	56/74 – 69/83 58/76 – 67/81	0.12 0.13
ϑ_200_	2.8 ccm 2.2 ccm	10/80 – 24/77 7/66 – 27/91	**<0.01** **<0.01**	− −	− −	− −	3.3 ccm 2.2 ccm	24/109 – 20/48 15/75 – 29/82	**<0.01** **0.02**	2.7 ccm 2.3 ccm	55/74 – 70/83 61/84 – 64/73	**0.049** **<0.01**
NT D_2ccm_	173.8 Gy 167.5 Gy	21/119 – 13/38 22/125 – 12/32	**0.03** **0.02**	− −	− −	− −	88.3 Gy 62.3 Gy	10/35 – 34/122 0/6 – 44/151	0.54 0.11	77.2 Gy 169 Gy	14/19 – 111/138 100/128 – 25/29	0.19 0.19
NT D_5ccm_	85.4 Gy 87.7 Gy	15/97 – 19/60 16/101 – 18/56	**<0.01** **<0.01**	− −	− −	− −	55.2 Gy 46.5 Gy	3/19 – 41/138 1/12 – 43/145	0.12 0.07	67.2 Gy 66.3 Gy	26/35 – 99/122 23/32 – 102/125	0.14 0.08
NT D_7.5ccm_	39.2 Gy 41.0 Gy	13/65 – 21/92 16/78 – 18/79	0.33 0.38	− −	− −	− −	50.4 Gy 50.0 Gy	21/101 – 23/56 21/119 – 23/56	**<0.01** **<0.01**	58.2 Gy 58.2 Gy	83/113 – 42/44 83/113 – 42/44	**<0.01** **<0.01**
B D_0.1cc__m_	− −	− −	− −	66.1 Gy 66.1 Gy	2/129–3/28 2/129–3/28	**0.02** **0.02**	− −	− −	− −	− −	− −	− −
B D_2ccm_	− −	− −	− −	59.3 Gy 59.3 Gy	2/128– 3/29 2/128 – 3/29	**0.02** **0.02**	− −	− −	− −	− −	− −	− −
B D_5ccm_	− −	− −	− −	58.6 Gy 58.6 Gy	2/128– 3/29 2/128 – 3/29	**0.02** **0.02**	− −	− −	− −	− −	− −	− −
CTV D_75_	92.9 Gy 113.3 Gy	22/120 – 12/37 27/141 – 7/16	**0.04** **0.04**	− −	− −	− −	67.1 Gy 65.2 Gy	4/22 – 40/135 3/18 – 41/139	0.14 0.12	66.6 Gy 66.3 Gy	13/19– 112/138 13/19 – 112/138	0.07 0.07
CTV D_90_	77.0 Gy 76.8 Gy	21/116 – 13/41 21/116 – 13/41	**0.04** **0.04**	− −	− −	− −	57.1 Gy 39.0 Gy	4/20 – 40/137 0/4 – 44/153	0.24 0.14	55.3 Gy 55.3 Gy	13/19– 112/138 13/19 – 112/138	0.07 0.07
CTV D_98_	61.0 Gy 60.6 Gy	12/74 – 22/83 12/73 – 22/84	0.06 0.07	− −	− −	− −	60.0 Gy 59.6 Gy	14/68 – 30/89 14/68 – 30/89	**0.03** **0.03**	47.5 Gy 47.2 Gy	17/24– 108/133 17/24 – 108/133	0.10 0.10
V_100,__CTV_	95.6 % 91.2 %	21/106 – 13/51 4/32 – 30/125	0.21 0.08	− −	− −	− −	95.5 % 91.4 %	24/101 – 20/56 5/23 – 39/134	0.07 0.30	95.0 % 94.9 %	63/84 – 62/73 64/85 – 61/72	0.06 0.07
DNR	0.29 0.28	8/45 – 26/112 9/55 – 25/102	0.27 0.12	− −	− −	− −	0.32 0.31	27/101 – 17/56 28/102 – 16/55	0.33 0.43	0.30 0.28	41/56 – 84/101 32/46 – 93/111	0.06 **0.02**
CTV vol.	11.8 ccm	12/88 – 22/69	**<0.01**	− −	− −	− −	10.2 ccm	11/70 – 33/87	**<0.01**	12.3 ccm	71/90 – 54/67	0.37

Using ACE_uniform_ calculations, all aforementioned correlations were found as well, although partly different separation thresholds were identified (for details, please see Table 2). However, ACE_uniform_ also showed significant associations of osteoradionecrosis and xerostomia with ϑ_200_ ≥ 3.0ccm (p = 0.04) and DNR ≥ 0.28 (p = 0.02), respectively, which were not observed for TG-43 calculations.

## Discussion

We reported clinical single-center results for the brachytherapy of oral cavity cancer as well as relations between clinical outcomes (efficacy and side effects) and corresponding water-based TG-43 and (to our knowledge for the first time in this regard) model-based ACE_uniform_ dose calculations. In this respect, various thresholds associated with increased toxicity were identified (Table 2), as for instance D_2ccm_ ≥ 173.8 Gy, D_5ccm_ ≥ 85.4 Gy, and D_7.5ccm_ ≥ 50.4 Gy for the oral cavity normal tissue, a CTV volume larger 10.2–11.8 ccm, a CTV D_90_ ≥ 77 Gy, and bone doses ≥ 58.6 Gy (all reported for TG-43).

With respect to osteoradionecrosis and soft tissue necrosis, Garcia-Consuegra et al.[34] reported similar dose constraints of 61 Gy for D_2ccm_ bone and 87 Gy for a tissue volume parameter depending on the ϑ_100_ size. Hence, our analysis confirmed a corresponding dependency on the size of total dose. For mucositis and xerostomia, particularly normal tissue D_7.5ccm_ ≥ 50.4 Gy and ≥ 58.2 Gy, respectively, represented significant risk factors to our knowledge not considered so far. Importantly all isodose volumes and the CTV volume studied in our analysis were associated with increased incidence of mucositis and soft tissue necrosis, concomitant with recent results revealing a link between soft tissue necrosis and CTV volume as well[34,35]. However, it has to be mentioned at this point that investigating relative isodose volumes may be of limited relevance if the underlying prescription doses vary (e.g., due to different fractionation schemes) or if the proportions of brachytherapy and EBRT on the total administered dose differ. Nevertheless, these parameters were also evaluated in the present work for the determination of corresponding thresholds, in particular to identify differences resulting solely from the choice of dose calculation algorithm and thus to sensitize for the corresponding potential clinical influence. In this respect, summing the absolute doses between brachytherapy and EBRT provided a more robust assessment of the dose-toxicity-correlations, which allowed a conservative estimation of the total dose to individual structures independent of the exact contribution of brachytherapy and EBRT to the overall treatment.

Moreover, surprisingly, we found a correlation of local recurrence with increased DNR values ≥ 0.31, which might be due to the fact that larger DNRs may result from prolonged dwell times at individual source positions potentially required for an adequate target volume coverage. This could reduce the treatment plan robustness again implant instabilities compared to more optimal catheter placements allowing sufficient dose coverage even with lower time weighting of source positions and hence DNR, which has to be investigated in further studies. However, it has to be noted that there is no dose-volume optimization technique that can correct a suboptimal implant geometry without resulting in extended high-dose areas and potentially increased toxicity, highlighting the importance of a high implantation quality. In this respect, brachytherapy of the oral cavity can be associated with a high level of complexity, which may require (depending on the clinical skills and experience of the involved physicians) interdisciplinary cooperations[36], e.g. with ENT surgeons. Nevertheless, the present work suggested a compliance with fixed dose constraints to limit toxicity, which was for the first time reported in our previous analyses[37] revealing for the pre-3D era correlations with local failure, osteoradionecrosis, and soft tissue necrosis for parameters of natural dose-volume-histograms. However, it is evident that unified constraints for image-guided brachytherapy of oral cavity cancer still have to be established, with the present work contributing to this important issue. In this respect, deformable image registration[38,39] for dose summation between brachytherapy and EBRT should be implemented to strengthen corresponding findings beyond a simple adding of EQD2 doses for individual dose parameters as conducted in the present work.

It should be noted that the dose thresholds determined differ between TG-43 and ACE_uniform_ and that the use of ACE_uniform_ led to statistically significant dose–response correlations that were not observed with TG-43. The results also differed substantially in some cases, particularly for high-dose areas, so the decision for either TG-43 or ACE_uniform_ was considered relevant. In this respect, model-based dose calculation appeared to be more relevant with respect to the OARs, whereas there were only limited changes to the evaluated target volume parameters. It has been shown by many studies, that while TG-43 yields reasonable dosimetry for treatments deep inside the body, tissue dose is overestimated close to air-tissue transitions or patient surfaces due to a lack of backscatter in the real scenario[8,40]. Nowadays, most clinical brachytherapy dose calculations are based on TG-43 representing the current standard[8,12], suggesting a larger relevance of the TG-43 related outcome analysis for the short term. However, concomitant with an improved establishment of MBDCAs and assuming that these predict doses actually delivered to the tissue better than water-based calculations, the thresholds and correlations obtained using ACE_uniform_ are considered to be of far-reaching long-term importance. This holds especially for the oral cavity region with tissue/material transitions to air and bones located (particularly in complex cases) very close to the CTV.

In our opinion, one aim of modern brachytherapy (and radiotherapy in general) is to perform dose calculations as accurately as possible. The use of MBDCAs, which explicitly takes into account tissue inhomogeneities and material transitions in the dose calculation, appears therefore to provide a clear benefit over the solely water-based TG-43 approach. In this respect, it has to be mentioned that the current GEC-ESTRO recommendation[1] for head and neck brachytherapy states that the “use of the TG-43 formalism is recommended, as published differences using Monte Carlo or a grid-based Boltzmann solver are not clinically relevant“. However, the references Peppa et al.[41] and Siebert et al.[42] used in this recommendation to justify this statement did only investigate differences between TG-43 and MBDCA regarding physical dose and radiobiological model parameters (which were found to be partly statistically significant but of low absolute size), but a correlation of MBDCA dosimetry to actual clinical outcomes was not performed. To the best of our knowledge, our analysis is the first to provide a corresponding investigation for head and neck brachytherapy, and revealed that there actually might be a relevance regarding the determination of dose thresholds associated with increased toxicity rates. Further studies by other authors/institution will have to validate our single-institution findings in the future. In the present work, we used ACE_uniform_ to reduce the impact of dental metal artifacts on dosimetry, taking a recent publication[17] into account that showed for various entities only small deviations from ACE calculations with explicit CT-number considerations. A comparison of the latter as well as further MBDCAs (e.g., Monte-Carlo simulations)[11] to ACE_uniform_ for oral cavity patients was beyond the scope of the present study, but further investigations should focus on this issue to evaluate the comparability of corresponding dosimetric results regarding clinical outcomes. Nevertheless, our work provides an important component for the increasing establishment of MBDCA in clinical practice. While, as mentioned above, almost the entire clinical experience in the field of brachytherapy is based on TG-43 calculations, the evaluation of corresponding dose-toxicity effects using MBDCAs is necessary to increase confidence in corresponding procedures, to demonstrate their advantages such as adapted dose constraints as shown in the present work, and thus to support the increasing clinical establishment.

A weakness of our manuscript is the heterogeneous patient collective comprising various treatment concepts. Analyzing differences between individual groups revealed deviations in the occurrence of side effects (e.g., enhanced mucositis and xerostomia incidence for patients receiving EBRT), but the reduced number of patients in these sub-groups had to be kept in mind. However, heterogeneity of cohorts in this respect is also widespread in literature, presumably due to the majority of institutions treating only a low number of corresponding patients in general[35]. Nevertheless, five-year overall survival and local-control rates of 60–78 % and 73–89 % have been reported for oral cavity brachytherapy in different clinical settings[6,15,35,43], which is in-line with our results of 71 % and 79 %, respectively. For soft tissue necrosis and osteoradionecrosis forming the most relevant toxicities, our presented rates of 22 % and 3 % were also comparable to further reports[8,15,44,45] yielding rates of 2–30 % and 2–4 % for image-guided brachytherapy, respectively. This is a particular improvement against the era prior to 3D image-guided treatment planning, where rates of up to 36 % for soft tissue necrosis[7,46] up to 10 % for osteoradionecrosis[7,47] at comparative local control rates were achieved. Image-guidance thus reduced the toxicity profile of brachytherapy in general, as also reported previously[8].

## Conclusion

In summary, our investigations found significant dependencies of clinical outcomes on the quality of dose distributions and dosimetric parameters, which should be considered in clinical routine. The selected dose calculation approach impacted corresponding correlations and also dose constraint values. This is particularly important for treatments close to material/tissue heterogeneities. The rationale of considering MBDCA calculations in clinical practice is the desire to perform dose calculations as accurately as possible and hence considering tissue heterogeneities and material transitions, which are not addressed using the solely water-based TG-43 approach. Therefore, future focus should be put on the increased establishment of MBDCAs into clinical practice and their impact on dose-outcome assessments. This will help to unify and specify dose constraints for clinical practice, and can finally ensure a high treatment quality to manifest brachytherapy as important treatment modality for oral cavity cancer.

## Declaration of Competing Interest

The authors declare that they have no known competing financial interests or personal relationships that could have appeared to influence the work reported in this paper.
